# An information theory account of cognitive control

**DOI:** 10.3389/fnhum.2014.00680

**Published:** 2014-09-02

**Authors:** Jin Fan

**Affiliations:** ^1^Department of Psychology, Queens College, The City University of New YorkFlushing, NY, USA; ^2^Departments of Psychiatry and Neuroscience, Icahn School of Medicine at Mount SinaiNew York, NY, USA

**Keywords:** cognitive control, information theory, uncertainty, entropy, frontoparietal network

## Abstract

Our ability to efficiently process information and generate appropriate responses depends on the processes collectively called *cognitive control*. Despite a considerable focus in the literature on the cognitive control of information processing, neural mechanisms underlying control are still unclear, and have not been characterized by considering the quantity of information to be processed. A novel and comprehensive account of cognitive control is proposed using concepts from information theory, which is concerned with communication system analysis and the quantification of information. This account treats the brain as an information-processing entity where cognitive control and its underlying brain networks play a pivotal role in dealing with conditions of uncertainty. This hypothesis and theory article justifies the validity and properties of such an account and relates experimental findings to the frontoparietal network under the framework of information theory.

## Introduction

The brain is constantly bombarded with more information from multiple sensory channels than it can process. A critical challenge that it must address is to ensure that only goal-relevant information reaches the level of focused attention. However, information that does not reach that level cannot and should not be fully excluded from ever reaching it because the information may have behavioral relevance. Therefore, there is a need for a dynamic control mechanism that permits the flexible allocation of resources to process subjectively important information. *Cognitive control* refers to processes that flexibly and adaptively allocate mental resources to permit the dynamic selection of thoughts and actions in response to context-specific goals and intentions (Posner and Snyder, 1975; Miller, 2000; Badre, 2008; Kouneiher et al., 2009; Solomon et al., 2009).

Behaviorally, cognitive control is studied by using tasks where there is an inherent conflict elicited by the stimuli or responses, for example, in Stroop (Stroop, 1935; MacLeod, 1991) and flanker (Eriksen and Eriksen, 1974) tasks. The magnitude of this *conflict effect* is indexed by the difference in reaction time (RT) and accuracy across conditions with and without competing stimulus dimensions (e.g., incongruent vs. congruent conditions in the color Stroop) or computations (e.g., color naming with or without word meaning processing). Conflict processing is usually accompanied by prolonged RT and increased error rate (e.g., Pardo et al., 1990; Carter et al., 1998; Botvinick et al., 1999; Carter et al., 2000; Leung et al., 2000; van Veen et al., 2001; Fan et al., 2005, 2003, 2007b, 2008b; Nee et al., 2007). Although conflict effects are typically used in the study of cognitive control, I argue that they constitute a special case of more general informational uncertainty. In what follows, I will take conflict to reflect a high level of entropy (average uncertainty) over competing choices, actions, or policies. This is to distinguish it from the corresponding uncertainty over states of the world that is resolved through perceptual inference. In other words, I will be focusing on the uncertainty about what to do in a given context—assuming that the context has been estimated or inferred.

The general neural circuitry underlying cognitive control is still not completely known, but activity in the anterior cingulate cortex (ACC) has been consistently demonstrated in functional magnetic resonance imaging (fMRI) studies [and also studies employing positron emission tomography (PET), electroencephalography (EEG), and other imaging techniques] involving tasks that invoke cognitive control. There is reliable functional activation of the ACC and dorsolateral prefrontal cortex (DLPFC) in tasks requiring the detection and resolution of conflict (e.g., Pardo et al., 1990; Botvinick et al., 1999, 2001; Carter et al., 2000; MacDonald et al., 2000; Fan et al., 2003, 2005, 2007b, 2008b; Liu et al., 2004). Consequently, at least two major theories of cognitive control relate ACC activity to the monitoring of conflict (Carter et al., 1998, 2000; Botvinick et al., 1999, 2001; MacDonald et al., 2000; Braver et al., 2001) or the resolution of conflict (Posner and DiGiralomo, 1998) (for other theories of ACC, see Ridderinkhof et al., 2007). Contrary to these theories, I argue that ACC, anterior insular cortex (AI), and other brain areas of the frontoparietal network process uncertainty, and will demonstrate that conflict is a special case of increased uncertainty.

## Information theory and cognitive control

### Definition of information: entropy, surprise, and entropy rate

Before I discuss the role of information theory in cognitive control, I will briefly review its key concepts. In Shannon's information theory (Shannon and Weaver, 1949), information is defined as entropy, a measure of uncertainty or freedom of choice when selecting a message (e.g., a sequence of symbols). The information entropy of a discrete random variable *X* that can take the possible events of {*x*_1_ … *x*_*n*_} is

(1) $$H(X)=E(I(X))=-\sum_{i\;=\;1}^{n}p(x_{i})\log_{2}p(x_{i})\;,$$

where *p*(*x*_*i*_) is the probability of event *x*_*i*_. Entropy is in units of bits, because of the base 2 logarithm. Information entropy quantifies the information contained in a message (a sequence) sampled from *X*, whereas *I(X)* is the information content. The *surprise*,

(2) $$I(x_{i})=-\log_{2}p(x_{i})\;,$$

quantifies the information conveyed by the occurrence of event *x*_*i*_. A low probability event has a high surprise measure.

In an event sequence, if events are predictable, the uncertainty of the events is low and thus the information entropy of this sequence is low. For example, a long sequence with a repeating series of events has an entropy of 0 bits, because every event is predictable.

The information entropy in the case of a RT task using two response alternatives with probabilities *p* and *q* = 1 − *p* is

(3) $$H=-\left(p\;log_{2}p\;+q\;log_{2}q\right).$$

**Figure 1:**
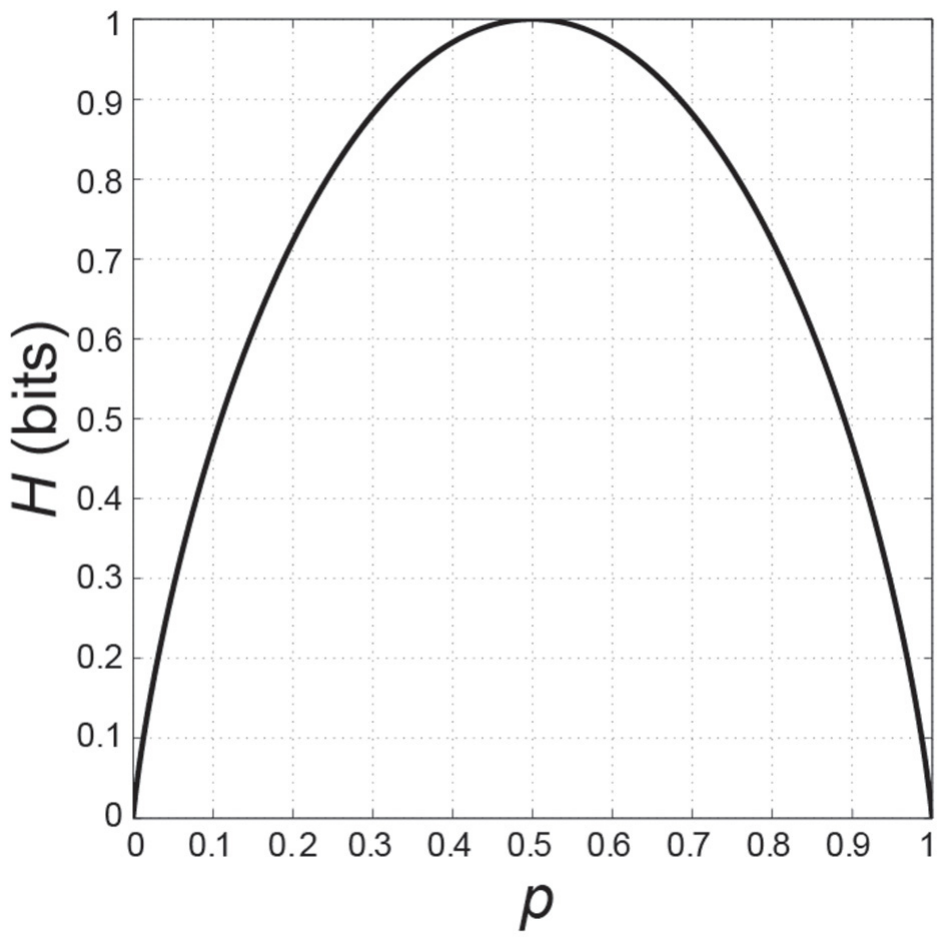
**Information entropy *H* as a function of the probability *p* in the case of two possible events**.

If we plot *H* as a function of *p*, it is an inverted U-function with *H* = 0 if *p* is 0 or 1, and *H* = 1, its maximum, when the probabilities of the two choices are equal, i.e., if *p* = *q* = 0.5 (Figure 1).

Suppose there are two events *x* and *y*. Let *p(i, j)* be the probability of the joint occurrence of events *x* and *y*. The entropy of the joint event is

(4) $$H(x,y)=-\sum_{i,j}p(i,j)\log_{2}p(i,j)\leq H(x)+H(y)\;.$$

That is, the entropy of a joint event is less than or equal to the sum of the entropy of the individual events, with equality only if the events are independent. This also implies that dependent events will have lower entropy, and thus less uncertainty, than independent events. This difference is, in fact, equal to the mutual information or relative entropy of the two events.

In this framework, information is processed via channels with limited capacity. The *channel capacity* is the upper bound on the amount of information that can be reliably transmitted (per unit time) with some arbitrarily small error probability. The *entropy rate* of a given channel is the average information transmitted per unit time. Specifically, it is the time density of the average information in a stochastic process. Finally, *channel switching* and *channel selection* are also essential, and there should be performance costs associated with these processes.

Entropy can be used to quantify both low- and high-level uncertainty. In digital computing, a bit is a binary digit, taking a value of either 0 or 1, as a basic unit of information storage and communication. The bit has been used to model uncertainty at the neuronal level (e.g., Quian Quiroga and Panzeri, 2009). In Shannon's information theory (Shannon, 1948), entropy can also represent higher-level uncertainty associated with selection of, for example, letters or words in a message.

### Information theory in cognitive science and neuroscience

Information theory has a long and distinguished role in cognitive science and neuroscience. The “cognitive revolution” of the 1950s, as spearheaded by Broadbent (1958) and Miller (1956), was highly influenced by information theory. Subsequent to the publication of Shannon and Weaver's book on information theory (Shannon and Weaver, 1949), many psychological studies demonstrated that RT in a key-press task is linearly related to the amount of information transmitted from the stimulus to the subject (Hick, 1952; Hyman, 1953) (see Attneave, 1959 for a review). Hick's Law (Hick, 1952), for example, states that RT is a logarithmic function of the number of response alternatives, implying a linear relationship between RT and input information in bits. However, the use of these kinds of models was complicated by experiments which showed that estimates of information processing capability differed depending on the response paradigm (Posner, 1966). With the development of cognitive psychology (Neisser, 1967), interest in relating information theory and RT waned as more emphasis was given to specific mental operations involved in task processing (e.g., Sternberg, 1969). As a result, quantitative analyses of the relationship between mental operations and the amount of information to be processed have not been fully developed.

With the development of neuroimaging techniques and theories (Posner and Raichle, 1994), it has become possible to specify the relationship between information quantities and brain activation, allowing us to fully study cognitive control of information processing. Two earlier papers speak directly to uncertainty, predictability and surprise in the hippocampus (Strange et al., 2005; Harrison et al., 2006) using fMRI. Other early examples include work on redundancy and efficiency (e.g., Barlow, 1961) culminating in notions like the principle of maximum mutual information or information transfer (e.g., Linsker, 1990). Over the past decade or so, this information theoretic approach to understanding perception and action has been cast in terms of Bayesian inference. One example is the free energy principle that has been used to account for a wide range of perceptual, attentional and behavioral faculties (Friston and Stephan, 2007). The free energy formulation is important here because the variational free energy is a proxy or bound on informational surprise (and the time average of this surprise is average uncertainty or entropy). This means that brain systems which minimize free energy serve to reduce surprise and average uncertainty (Friston and Stephan, 2007; Friston, 2010; Friston et al., 2012). There are a number of schemes that have been proposed to minimize average uncertainty (free energy). The most popular and neuronally plausible scheme is known as Bayesian filtering or predictive coding (e.g., Bastos et al., 2012). In the setting of cognitive control and decision theory, this quintessentially information theoretic approach makes some strong predictions about the functional anatomy of choice behavior that highlights the role of systems like the ACC (and prefronto-striatal loops) in minimizing surprise. We will see examples of this later.

### Information estimates of conditions in tasks involving cognitive control

Examining uncertainty across different paradigms used to study cognitive control, at both the individual event and event sequence levels, will lead to a fundamental understanding of cognitive control and its related brain activity (Koechlin et al., 2003; Yoshida and Ishii, 2006; Koechlin and Hyafil, 2007; Koechlin and Summerfield, 2007; Badre, 2008). Studies of cognitive control employ tasks in which information content, frequency of appearance, or processing rate are manipulated. These quantities map directly to entropy, surprise, and channel capacity respectively. I will show how these values can be estimated from cognitive control tasks that involve competition between stimulus or response dimensions, that rely on infrequent events, and that require increasingly complex mental algorithms.

In the most commonly used cognitive control tasks, such as color Stroop and flanker tasks, the difference between conditions, usually attributed to cognitive load or conflict, can be defined by a difference in relative uncertainty. The conflict effect generated by an interfering dimension, e.g., from the word meaning in a color-word Stroop task (MacLeod, 1991; MacDonald et al., 2000; Fan et al., 2003), from the flankers in a flanker task (Eriksen and Eriksen, 1974; Cohen and Shoup, 1997; Botvinick et al., 1999; Casey et al., 2000; Fan et al., 2003), or from a global or local feature in a global/local selective attention task (Weissman et al., 2003) can be estimated as an uncertainty difference of up to 1 bit between conflict and no-conflict conditions. In the color Stroop task, entropy can be calculated from the number of possible responses. This level of uncertainty is not the same across neutral and incongruent conditions. In the neutral condition, the word meaning does not interfere with the color of the word, and only one response can be mapped to each stimulus. However, in the incongruent condition, the word meaning is inconsistent with the color of the word. This incongruency increases the number of possible responses mapped to each stimulus to 2, and corresponds to an increase in entropy of 1 bit. This difference is often measured as a RT difference of ~100 ms. However, if interference from the word is suppressed by visually blurring the letters or some kind of manipulation (e.g., Raz et al., 2002, 2005), the relative uncertainty difference should be less than 1 bit.

For flanker tasks, let 0 and 1 represent left and right pointing arrows, respectively. Then, congruent trials are represented as 00000 and 11111. Incongruent trials are represented as 11011 and 00100. The underlined center digit is the target. Because of information reduction (Posner, 1964) or perceptual grouping (Wagemans et al., 2012) even without attention (Moore and Egeth, 1997), arrows pointing to the same direction can be grouped into reduced representations as 0 or 1 and 10 or 01. If subjects can filter out the irrelevant flanker digit, the predicted uncertainty for the congruent and incongruent conditions is the same 1 bit, with no difference between conditions. However, if subjects cannot filter out the flanker, each stimulus in the incongruent condition indicates an additional possible response, and increases the uncertainty to 2 bits. Then, the difference between the incongruent and congruent conditions is 1 bit. Top-down attentional control can be used to suppress the flankers, although experimentally the suppression is imperfect and does not occur on every presentation. Therefore, the average uncertainty difference between the incongruent and congruent conditions should be less than 1 bit. The corresponding RT difference is typically between 50 and 150 ms, with some exceptions associated with size and shape of the arrows, distance between flanker and target, or procedural variations (Weekes and Zaidel, 1996).

The central aspect of these paradigms is interference between the prior or prepotent response and responses called for on the basis of sensory evidence. The ensuing conflict (uncertainty or high entropy over plausible responses) can be seen under a Bayesian perspective. In other words, if we associate the prepotent response with a prior distribution, the posterior distribution (given incongruent sensory cues) will induce a high entropy distribution over possible responses associated with conflict. One can see clearly how the use of interference between priors and sensory likelihood provides an elegant paradigm to experimentally manipulate levels of conflict in this framework.

Paradigms investigating the oddball effect, Go/No-Go performance, or task switching involve examining responses to a stimulus type with a low probability of occurrence in a series of high-probability events. The amount of uncertainty to be processed in these tasks can be estimated using the surprise and entropy equations. For a Go/No-Go task, assume that probabilities for Go and No-Go trials are 0.80 and 0.20, respectively. The *surprise* (Equation 2) for Go and No-Go trials can then be computed as 0.32 and 2.32 bits. Therefore, the difference in information conveyed by the occurrence of Go and No-Go events can be quantified as the 2-bit difference between these two surprise values. Now assume that there is an additional condition where the probabilities for Go and No-Go trials are both 0.50. The difference in entropy between these two conditions (sequences) can then also be examined (Equation 1). The entropy for the former condition is approximately 0.70 bits, and is 1 bit for the latter. The ACC and AI activation defined by a No-Go > Go contrast (e.g., Schulz et al., 2004) may be related to the 2 bit increase in *surprise* instead of the usual explanations of inhibitory or response control.

In one type of task switching study, participants are asked to alternate between performing two tasks. On the trials when the task is switched, participants show increased RT and decreased accuracy. The frequency of switch trials is usually much lower than non-switch trials (e.g., in Dove et al., 2000). Recall that based on Equation 2, low probability of occurrence is associated with a large surprise value. Even if the number of trials for each task are equal (e.g., in Rushworth et al., 2002), or the order of the task blocks is unpredictable (Yeung et al., 2006), the switch trials themselves are infrequent and the effects for the first few trials after switching can be explained by the increase in surprise, rather than the switch between tasks *per se*. In addition, channel switching (e.g., alternating left and right responses, compared to repeating one response) and selection (e.g., changing the response from auditory to visual modality) should also be related to a performance cost.

**Figure 2:**
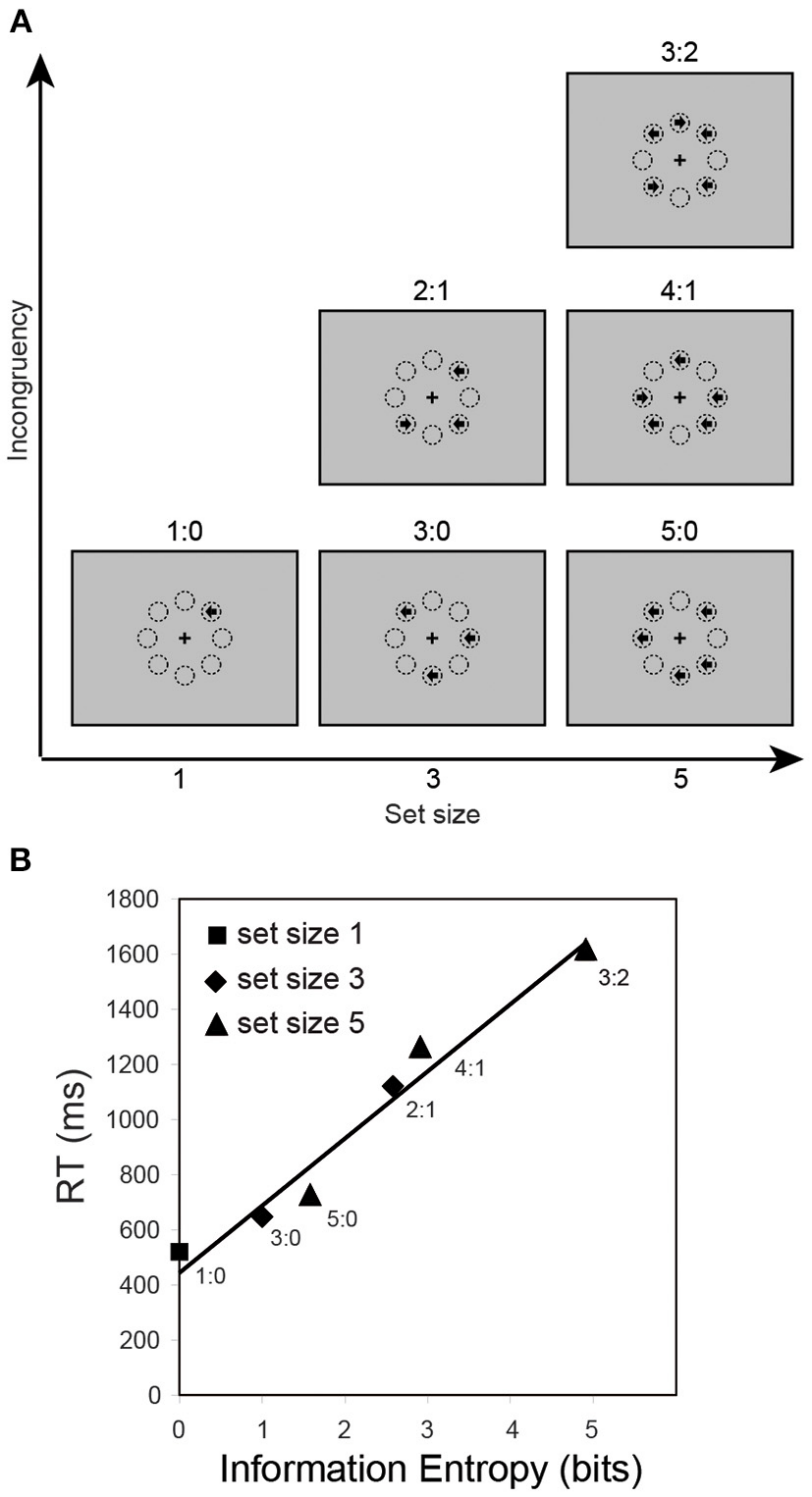
**The majority function task and reaction time as a function of information entropy. (A)** In this task, arrows with set sizes of 1, 3, or 5 are randomly presented at 8 possible locations arranged in an octagon centered on a fixation cross. The arrows point either left or right, and are presented simultaneously. The participants' task is to indicate the direction in which the majority of arrows point. For example, if three arrows are presented, and two point to the left and one to the right (see the “2:1” panel in the “Set size 3” column), the correct response should be “left.” The eight circles illustrate the locations and are not displayed during the experiment. The label for each of the 6 conditions is the ratio of the numbers in each category. **(B)** Reaction time (RT) as a function of information entropy (estimated based on a group search strategy).

In addition to uncertainty due to dimensional competition in the stimuli or manipulations of stimulus frequency, there is also a need to consider uncertainty due to the algorithms of mental operation for tasks that involve higher level stages of uncertainty processing (Bach and Dolan, 2012), such as the majority function task (MFT, Fan et al., 2008a, 2014; Wang et al., 2011). In this task, participants are shown a number of left/right arrows and asked to indicate the direction in which the majority of the arrows are pointing (Figure 2A). Set size (1, 3, or 5 arrows) and congruency (the ratio of the number of left/right arrows) are varied across and within blocks of trials, respectively. A majority function can be computed that outputs 1 if and only if more than half the inputs are 1s. For example, given three input bits *x, y, and z*, the majority can be computed based on the formula *majority*(*x*, *y*, *z*) = (*x* ∧ *y*) ∨ (*y* ∧ *z*) ∨ (*x* ∧ *z*) (Wang et al., 2011). In a flanker task, the center arrow and the surrounding arrows are explicitly defined as target and task-irrelevant distracters. However, in the MFT, all arrows displayed in a set are possible task-relevant. Although the input information to be processed varies across conditions, the response is always only 1 bit (two alternatives, left/right). Using RT as a measure of cognitive control, the information entropy based on different searching algorithms was estimated. RT was best predicted by a grouping search algorithm involving sampling and resampling of the inputs to find a coherent majority sample, compared to alternative algorithms (i.e., exhaustive or self-terminating search) (Fan et al., 2008a). The entropy estimates are 0, 1, 2.58, 1.58, 2.91, and 4.91 bits corresponding to 1:0, 3:0, 2:1, 5:0, 4:1, and 3:2 ratio conditions, and these estimates correspond to an increase in RT (Figure 2B).

The uncertainty of the information to be processed is encoded by the grouping search algorithm rather than the outcome. Let us assume that human subjects apply this grouping sampling strategy by searching for a congruent sample with a majority grouping size (e.g., 1, 2, and 3 for set size 1, 3, and 5 respectively). For set size 1, only 1 arrow needs to be scanned. For set size 3, if all 3 arrows point in the same direction, only 1 grouping attempt needs to be made with 2 arrows being scanned; and if only 2 arrows point to the same direction, on average there will be 1 successful grouping out of every 3 attempts. Therefore, 6 arrows need to be scanned, which is the product of 3 grouping attempts and group size of 2 arrows. Similarly, for set size 5, on average 3, 7.5, and 30 arrows need to be scanned, respectively, for the conditions in which 5, 4, or 3 arrows point to the same direction, because on average 1, 2.5, 10 grouping attempts will be needed to reach a congruent group. If we use the majority group size as the information unit, assuming that each sampled group is equivalent to one unit of information, the information to be processed is log_g_ (s), where the base g represents the group size and s is the number of arrows to be scanned. To convert this measure to bits (i.e., from base g to base 2), it is multiplied by log_2_ (g). Therefore, the computational load is log_2_ (g) • log_g_ (s), which is equivalent to log_2_ (s). The sensory information in the MFT may not automatically accumulate over time, as in temporal integrator models (see Kayser et al., 2010; Bach and Dolan, 2012) based on the Newsome motion coherence task (Newsome et al., 1989; Kiani and Shadlen, 2009).

## Brain networks involved in cognitive control

### The involvement of the ACC in uncertainty processing

**Figure 3:**
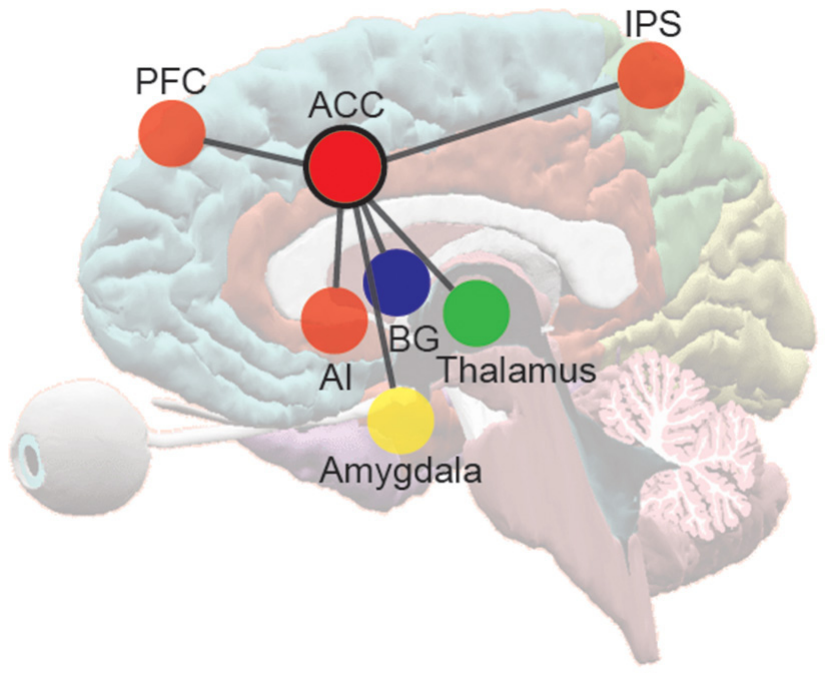
**The frontoparietal network and the pivotal role of the ACC in information processing and response across domains**. ACC, anterior cingulate cortex; AI, anterior insular cortex; BG, basal ganglia structures; PFC, prefrontal cortex; IPS, areas near and along the intraparietal sulcus.

After quantifying the uncertainty under different conditions in a range of tasks used to study cognitive control, I attribute differences in RT and error rate across conditions in these tasks to changes in uncertainty. I also attribute task-related differences in ACC activity to these uncertainty differences. The ACC is the anterior portion of the cingulate gyrus, and is located around the genu and anterior third of the corpus callosum. It is generally considered to be a frontal limbic neocortical field and is connected with the prefrontal and parietal cortices, the primary motor cortex, and the frontal eye fields (FEF). It also receives substantial input from midline and intralaminar thalamic nuclei, and from the amygdala (Vogt and Pandya, 1987). All efferents and afferents to and from the ACC travel via the cingulum bundle (Vogt and Gabriel, 1993). Coupled with other limbic and neocortical areas such as the AI, basal ganglia (BG) structures, the frontoparietal regions including the prefrontal cortex, and the parietal cortex, the ACC plays a crucial role in sensation and perception, executive control of attention (Posner and Petersen, 1990), emotion, social cognition, and response selection, preparation, and execution (Frith et al., 1991; Paus et al., 1993) (Figure 3).

Numerous tasks with no conflict effect also activate the ACC. Theories that strongly link conflict detection and resolution to ACC activity cannot explain these findings (see also Yeung, 2013). Activation of the ACC (and the AI) is associated with tasks involving salience and the oddball effect (Seeley et al., 2007; Sridharan et al., 2008), stimulus presentation frequency (Braver et al., 2001; Carreiras et al., 2009), violations of repeating patterns or sequences (Huettel et al., 2002; Ursu et al., 2009), decision making (Critchley et al., 2001; Ullsperger and von Cramon, 2004; Walton et al., 2004; Zysset et al., 2006; Behrens et al., 2007; Pochon et al., 2008), volatility of the reward environment (Behrens et al., 2007), and voluntary risk taking (Rao et al., 2008). Selection of random free movements compared to making predetermined movement is associated with increased regional cerebral blood flow (rCBF) in the ACC and motor areas (Deiber et al., 1991). The ACC is also engaged during tasks with a low-frequency of responses (Braver et al., 2001) and is activated more during random sequences (i.e., high uncertainty) than during fixed sequences (i.e., low uncertainty) (Koechlin et al., 2000). Increased ACC and DLPFC and decreased posterior cingulate cortex (PCC) rCBF are related to willed action independent of modality (Frith et al., 1991). The ACC neurons of macaques are more active while searching for new sequences of visual stimuli than while responding to a repeat sequence (Procyk et al., 2000). Furthermore, lesions to the ACC in macaques impair the ability to integrate current and past information to guide behavior (Kennerley et al., 2006). In all of these studies, the task conditions involve manipulations of uncertainty (Platt and Huettel, 2008; Rushworth and Behrens, 2008), possibly at different processing levels (Bach and Dolan, 2012).

Although ACC activity has also been linked to error processing (Kiehl et al., 2000), the ACC is activated in tasks involving processes beyond error detection. It is activated by stimulus novelty (Berns et al., 1997), by the violation of the regularity of stimulus sequences without subject awareness (Ursu et al., 2009), under conditions where errors are most likely to occur (Carter et al., 1998; Braver et al., 2001), and during error evaluation (Magno et al., 2006). Taken together, all these studies imply a consistent relationship between ACC activity and uncertainty, rather than errors *per se*.

Task-related increases in the effective connectivity of the ACC have been identified using fMRI time-series data. One study showed that the ACC modulates Heschl's gyrus and superior temporal gyri in an auditory oddball task, and influences the striate cortex in a visual oddball task (Crottaz-Herbette and Menon, 2006). This study supports the theory that the ACC exerts cognitive control by enhancing the processing of sensory signals for target stimuli to facilitate further processing. It further extends the finding of increased task-related synchronous activity between the caudal ACC and the primary and supplementary motor areas, supporting the hypothesis that the ACC directs cognitive control by modulating activity in diverse cortical regions (Posner and Raichle, 1994).

This enhancing suggests that the ACC is in a position to nuance the conflict by changing the entropy or uncertainty of choice probabilities. Mathematically, this corresponds to changing the precision of beliefs about which option is chosen. A simple way of seeing this is to consider the ubiquitous softmax response model of some value function—here the probability of making a particular choice. The softmax parameter known as precision or sensitivity sets the precision or fidelity of the choice by reducing the entropy of the conflict. In other words, a high precision will make the most likely option much more likely relative to competing options thereby minimizing uncertainty. Notice that this enables the brain to control its own expected uncertainty through optimizing the precision of beliefs. It may be that the ACC plays a special role in this important aspect of optimally reducing uncertainty (but see below). Indeed, current formulations of surprise or free energy minimization focus on interactions between the prefrontal cortex and striatum in optimizing the precision of beliefs about options or policies, where it is possible that the precision is signaled by neuromodulatory transmitters such as dopamine. Crucially, the descending connections from the ACC may play a pivotal role in optimizing the precision and thereby reducing uncertainty about future choices to an optimal level. This framework can be cast in terms of free energy minimization as described in the paper on the anatomy of choice by Friston et al. (2013).

### A general role of the ACC in information processing speed

Animal and human lesion studies have demonstrated evidence arguing against the necessity of the ACC in conflict processing. Lesions of the ACC and DLPFC in monkeys have shown no modulation of the magnitude of the conflict effect, suggesting that this effect may also be mediated by other brain structures (Mansouri et al., 2007). Humans with ACC lesions do not exhibit measurable current-trial conflict processing deficits (Fellows and Farah, 2005; di Pellegrino et al., 2007) (but do show a reduced context effect of the previous trial, Sheth et al., 2012). Two human lesion studies with relatively large sample sizes have also failed to find an effect of ACC damage on the conflict effect (Vendrell et al., 1995; Stuss et al., 2001). If the ACC is involved in conflict processing, then why do conflict effects not increase following ACC damage?

Contrary to existing theories that conflict processing is the main role of the ACC in cognitive control, I theorize that the ACC plays a general role in information processing speed, which is as a function of uncertainty. A typical assumption in studies of cognitive control is that the conflict effect and overall RT are orthogonal. However, a significant positive correlation between the magnitude of the conflict effect and overall RT can be consistently observed (Fan et al., 2002, 2007a, 2009; Wang and Fan, 2007). Further, relatively long overall RT has been found in patients with schizophrenia when performing tasks requiring conflict processing (Krieger et al., 2005), and is selectively associated with a greater conflict effect in patients with schizophrenia (Wang et al., 2005) and in children with dyslexia (Bednarek et al., 2004). This significant correlation may indicate that common factors or brain structures affect both conflict processing and overall response speed.

Some animal and human lesion studies have used ratio scores to index the conflict effect, calculated as the RT difference between conflict and no-conflict conditions divided by overall RT (or RT under no-conflict conditions) to partial out effects related to overall response speed. If the size of the conflict effect depends on the overall RT, however, this normalization would mask real differences in the conflict effect between groups. Human lesion studies have shown that the overall RT of subjects with ACC lesions is significantly longer than that of control subjects (Vendrell et al., 1995; Fellows and Farah, 2005; di Pellegrino et al., 2007). Lesions in the ACC did not produce selective changes in the conflict effect, but increased RT in both conflict and no-conflict conditions. ACC plays an important role in cognitive control demands, as indexed by RT [cf. the “time on task” account (Grinband et al., 2011) for a related but different theory]. Experiments that do not manipulate conflict show ACC activity when there is an increase in RT. Conflict-related theories fail to account for this evidence. The challenges to current theories of cognitive control can be addressed by the information theory account.

### Beyond the ACC: the frontoparietal network for cognitive control

The ACC contains a class of neurons called spindle neurons or von Economo neurons (VENs). VENs are very large, bipolar, vertical fusiform cells and have only been found in humans (Nimchinsky et al., 1995), great apes (Nimchinsky et al., 1999), some cetacean species (Hof and Van der Gucht, 2007), and elephants (Hakeem et al., 2009). VENs are most abundant in humans and are primarily found in clusters in layer Vb of the ACC, with highest densities in areas 24b and 24a. They are also found in a cytoarchitectonically distinct region located in the AI (Von Economo and Koskinas, 1925; Nimchinsky et al., 1995, 1999). VENs are projection neurons approximately 4.6 times the size of neighboring pyramidal neurons. The localization of VENs to specific functional regions suggests an integral role of these neurons in corresponding functions. Based on their laminar location, VENs are likely to have widespread connections with diverse parts of the brain (Allman et al., 2001). Because of their potential connectivity and their large axonal diameters, VENs are well suited to provide a fast relay of signals derived from information processed in the ACC to other prefrontal and temporal limbic areas. Thus, they can support rapid integration of input from spatially distinct functional regions as well as a quick assessment of unpredictable, rapidly changing, and complex inputs (Allman et al., 2005; Fan et al., 2011).

Recent research has started to reconsider ACC function within a larger network underlying cognitive control. This frontoparietal network includes the FEF, supplementary eye field, ACC and AI, middle frontal gyrus (MFG), the intraparietal cortex (IPC) near and along the intraparietal sulcus (IPS), and superior parietal lobule (Hopfinger et al., 2000; Kastner and Ungerleider, 2000; Corbetta and Shulman, 2002; Rossi et al., 2009). It is worth noting that the frontoparietal network's implementation of cognitive control may fulfill certain properties of modular systems (Fodor, 1983; Barrett and Kurzban, 2006), with distinct regions having their own specialized function.

Although the ACC and AI are commonly co-activated, they may have a functional dissociation with regards to uncertainty processing. The ACC can be considered the limbic motor cortex and the insular cortex can be viewed as the limbic sensory cortex (Craig, 2009). In this framework, the ACC plays an important role in executive control (voluntary, top-down) of responses. The AI, onto which highly processed sensory inputs converge (Flynn et al., 1999), integrates those inputs (automatic, bottom-up) in relation to the uncertainty of the bodily feeling state (Singer et al., 2009). Although not discussed in this article, the functional commonalities and specifications of the subdivisions of the ACC (Bush et al., 2000; Fan et al., 2008a; Nee et al., 2011) and AI (Kurth et al., 2010), and the dissociation between them (Gu et al., 2010; Medford and Critchley, 2010; Menon and Uddin, 2010; Sterzer and Kleinschmidt, 2010) also need to be considered to fully understand their roles in the network. The functional dissociation of the ACC and AI has recently been demonstrated under task conditions different from those mentioned here (Gu et al., 2010, 2012, 2013b).

The roles of other regions in the frontoparietal network are also crucial to cognitive control. It has been suggested that the frontoparietal regions of the FEF and IPS are related to both top-down, goal-driven and bottom-up, stimulus-driven aspects of cognitive control (Knight, 2007), and that the IPS modulates the activity of regions related to early visual inputs (Rossi et al., 2009). The lateral intraparietal area (LIP) of the IPS, the FEF, and the superior colliculus (SC) contain spatially restricted visual receptive neurons selectively responsive to behaviorally relevant objects of visuospatial input. It has been proposed that these areas encode the salience of objects with attentional weight (Gottlieb, 2007).

I propose that all key regions in the frontoparietal network activate as a function of demands for cognitive control, for both bottom-up and top-down information transmission and modulation, with their specific roles similar to those described in recent work (Anderson et al., 2008; Wang et al., 2010). The notion that the ACC is involved in the modulation and selection of options (or their underlying cues) is important in relation to the information theoretic characterization based upon precision. Note that if the ACC is involved in optimizing precision, then the ultimate effects have to be of a multiplicative or modulatory sort—in the sense that the precision of beliefs does not change their content just their influence.

I theorize that the frontoparietal network (ACC and AI being key structures in this system) for cognitive control facilitates rapid information processing and transmission under conditions of uncertainty. This system is involved in the cognitive control of information transmission and integration across specialized cortical and subcortical regions. The ACC and AI consistently process the uncertainty that is essential to the initiation of cognitive control (Fan et al., 2014), thus largely determining the efficiency of information processing. Greater activation of this system, especially in ACC and AI, is associated with cognitive control under a high computational load and a high information processing and transmission rate. In addition, when the computations in the ACC and AI become more involved in channel switching and selection, competition, and high rate transmission of information, there should be concomitant increased activity and connectivity of the ACC and AI.

## Testing the frontoparietal network as an information processing entity

Information theory characterizes the uncertainty of a communication system which has three essential parts: information source, channel, and destination (Shannon, 1948), each of which can be affected by noise. I conceive of the cognitive processing stream as such a communication system where the frontoparietal network serves as an integrative interface between input and response. Inputs to the processing stream from information sources are selectively routed through channels to one or several output destinations under the guidance of the cognitive control. This interface dynamically handles information uncertainty and prioritizes the transmission of one or several specific sources to output destinations for further processing. It may be implemented through the dynamic interconnections between the cortical and subcortical structures linked to the frontoparietal network. If any brain regions are such an information-processing entity, I propose that it must demonstrate three properties: *functionality*, *specificity*, and *capacity*. I will present some preliminary evidence that the frontoparietal network exhibits these properties.

**Figure 4:**
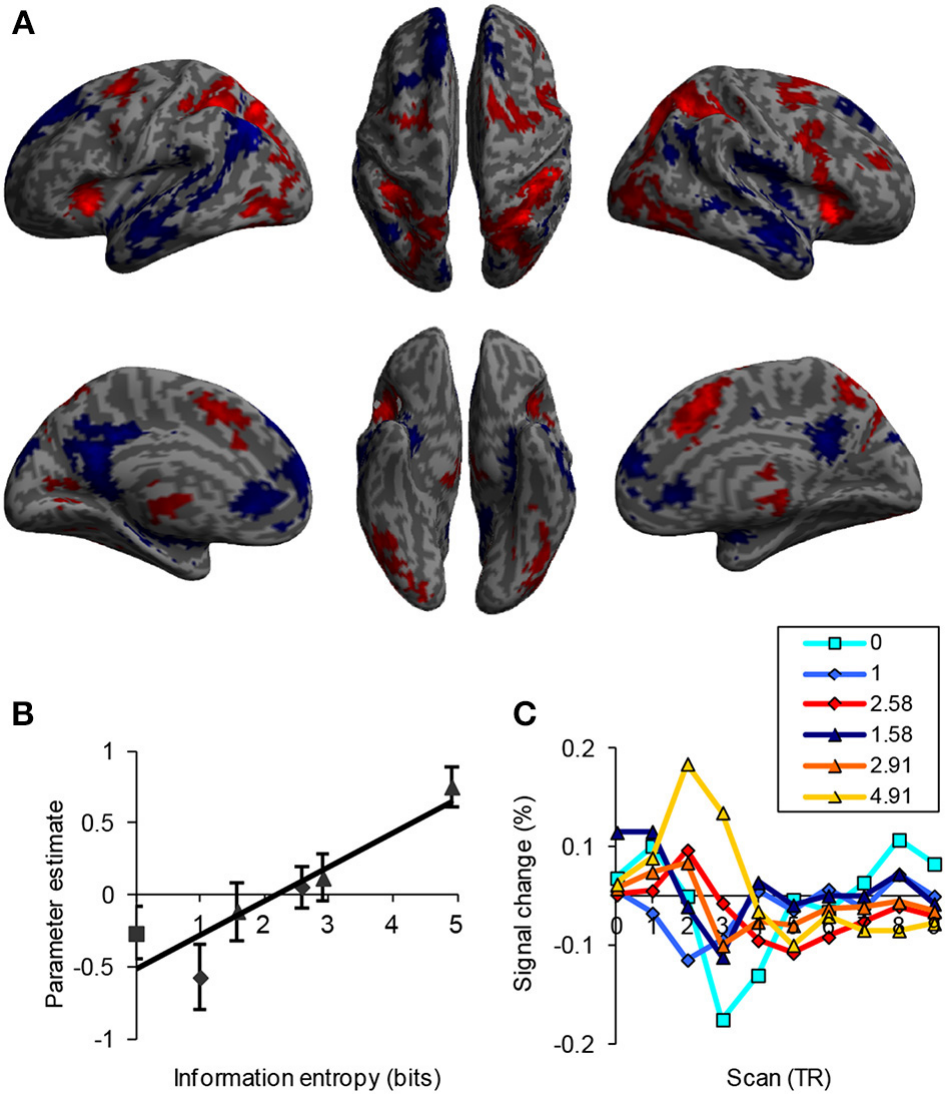
**Brain activation as a function of information uncertainty. (A)** Activation (in red) and deactivation (in blue) as a function of uncertainty (in bits). **(B)** Plot of beta value as a function of uncertainty. **(C)** Hemodynamic response as a function of uncertainty. *TR* = 2.5 s.

*Functionality* refers to the increase in activity of brain regions corresponding to an increase in uncertainty. Early information theory studies demonstrated a linear relationship between RT and information entropy (Hick, 1952; Hyman, 1953), indicating higher cognitive load with higher entropy. Therefore, the activity and connectivity of the frontoparietal network in the cognitive control of uncertainty processing should have a positive monotonic relationship with uncertainty, which is reflected by computational load, determined by both the amount of input information and the algorithms of the mental operations involved. In a recent study, the neural activity of the frontoparietal network was examined as a linear function of information uncertainty (Fan et al., 2014). A positive association between activity in those brain regions and uncertainty as measured by the MFT was demonstrated (Figure 4), supporting the functionality of the frontoparietal network in cognitive control. In contrast, regions of the default mode network were deactivated as a function of uncertainty. Although these regions may play an important role in cognitive control, they do not exhibit the property of functionality and thus do not process uncertainty as the frontoparietal network does.

*Specificity* refers to the modality-independence of the brain regions in the processing of uncertainty when cognitive control is needed. In another recent study, the specificity of the frontoparietal network with regard to information uncertainty was tested in a visual and an auditory fMRI experiment (Lee and Fan, in preparation). Participants were given sequences of single stimuli that came from a set of two classes. In the visual experiment, the stimuli could be left- or right-pointing arrows; in the auditory experiment, the stimuli could be low- or high-frequency tones. This study made no manipulations of conflict, and only varied the relative probability of the presentation of stimulus classes. Increases in activity of the frontoparietal network were associated with increases in entropy and surprise, and this specificity of the neural response was the same for both presentation modalities.

Finally, *capacity* refers to the maximum amount of information a channel can transmit per unit time. The limited capacity view was central to early views of attention that were influenced by information theory (Broadbent, 1958) and I similarly propose that the frontoparietal network has limited capacity. When the required processing rate exceeds the peak limitation, or when the power of noise in the channel increases, processing efficiency (as measured by both RT and accuracy) decreases. I hypothesize that there is a central bottleneck that determines the capacity of cognitive control. Based on Shannon's estimation of information processing in reading, cognitive control may have a capacity with an upper limit of ~50 bps. This may be an overestimation because language contains redundant information. Establishing this upper limit is an open research question.

## Comparisons with other accounts of cognitive control

To justify the validity of the information theory account of cognitive control, that is, the extent to which the concepts of this account can explain the empirical data, I will compare and contrast this account to other popular accounts of cognitive control in the literature.

### The conflict and context effects

The ultimate goal or the function of cognitive control is to reduce uncertainty. The information theory account is a general account of cognitive control. As established earlier, conflict is only one type of uncertainty increase, and this account extends its predictions beyond the accounts of conflict theories. Popular models strongly relating the ACC to conflict effects have limitations in their ability to explain the underlying information processing, and present only a special case of the information theory account.

The discrepancy between human studies showing conflict-related ACC activation and monkey studies failing to find ACC response to conflict monitoring has been attributed to differences of methodology used in different species [e.g., ACC and subdivision functional neuroanatomical differences, blood-oxygen-level dependent (BOLD) signal of population neurons vs. single-unit activity recording, task/response differences] (Cole et al., 2009). Other alternative interpretations have also been proposed (Schall and Emeric, 2010), because neurophysiological studies of the macaque ACC have shown consequence (error and reward) related signals but no conflict-monitoring related activity enhancement (Ito et al., 2003; Nakamura et al., 2005; Emeric et al., 2008). However, the structure of the tasks in terms of probabilities of specific events has not been systematically examined. ACC neurons have been shown to encode reward probability information at both choice and outcome epochs (Kennerley et al., 2011). In another study also using a probabilistic choice task (Hayden et al., 2011), dorsal ACC neurons of macaques showed enhanced response to the surprise from the outcome regardless of valence, even though there was no explicit conflict. These results support the idea that ACC activation in human neuroimaging studies is associated with the surprise resulting from less frequent conditions (e.g., error, with low probabilities). Therefore, comparing a high probability event with conflict to a low probability event without conflict, the ACC neurons may not fire more under the former condition, because firing rate increase depends on whether there is an increase in entropy.

The “context effect” (Gratton et al., 1992), or the conflict adaptation effect (Mayr et al., 2003), describes the reduction in RT to an incongruent flanker trial following another incongruent flanker trial. The effect can be explained as a reduction in uncertainty through two possible mechanisms. One involves an increase in cognitive control triggered by the context of the previous trial. In this situation, the increased cognitive control effectively filters out the flankers and reduces the uncertainty from the flankers. Another attributes the reduction in RT to exact repetitions of stimuli. In this case, there is no change in context or response, which corresponds to a low entropy scenario.

### Reinforcement learning and cognitive control

The information theory account is consistent with the account that ACC involves reinforcement learning and control in supporting the production of goal-directed action (Alexander and Brown, 2011), and the two make similar predictions across several different experimental paradigms. However, the information theory account attributes the activation of the frontoparietal network to the fundamental increase in uncertainty, while reinforcement learning accounts attribute the same activation to differences between a prediction signal and the actual outcome.

The predicted response outcome model of the ACC (Alexander and Brown, 2011; Nee et al., 2011; Brown, 2013) builds on the dominant conflict monitoring model (Carter et al., 1998; Botvinick et al., 2001) to account for ACC activation when there is no apparent conflict or error. It adds a key function of detecting not only the error likelihood but also the absence of an expected outcome (Brown and Braver, 2005). The model generates a large signal when a predicted event fails to occur. Situations that elicit this discrepancy also are high in surprise, due to the presence of low-probability events, and so this model makes similar predictions to the information theory account under these conditions. The predicted response-outcome model involves a single comparison between prediction and outcome per trial. An algorithm performing multiple comparisons best accounts for the data from the MFT, and the information theory account explains these multiple comparisons through activity in the frontoparietal network.

A different reinforcement learning model of the ACC (Holroyd and Coles, 2002) focuses on error-related negativity (ERN). The ERN generated in the ACC is highly sensitive to various sources of error information and depends on the mesencephalic dopamine system. It is not elicited by the error, but rather by error detection and the use of errors to prevent future errors. If we consider the ERN as a result of uncertainty during reinforcement learning, which is supported by the observation that the ERN can be also elicited with a correct response, the reduction of uncertainty will eventually bring the uncertainty to a stable state (i.e., error rate), and the uncertainty of that state can be quantified by surprise. Underlying the conflict and action selection models is the uncertainty requirement for cognitive control, independent of the source of the uncertainty.

Error and prediction error-related ACC activity is due to the increased surprise value of a trial or conditions that lead to more errors, rather than the error *per se*. It would be ideal to separate the effect of increased uncertainty from error-related effects. However, it is almost impossible to find a condition of high uncertainty that elicits perfect performance. In addition, the presence of an error or an unexpected outcome itself is usually a low probability event, which constitutes a high surprise value (Equation 2) and thus activates ACC and AI. Recently, the dorsal ACC has been proposed to integrate information about reward and costs in order to estimate the expected value of control (Shenhav et al., 2013). However, given that simple manipulation of stimulus or response probability can activate the ACC, this activity is related to the general computation of uncertainty, rather than specifically for the estimation of the expected value of control.

As described, information theory quantifies uncertainty at the sequence and event type level using entropy and surprise values, respectively. However, prediction and evaluation at the individual trial level, independent of context, and the associated information processing in the ACC should also be considered. It has been argued that ACC activation reflects not only the overall likelihood of an error in a trial (which can be quantified as surprise) but also the within-trial difference in the need for control (Anderson et al., 2008). Recently, it has been shown that the ACC predicts trial-by-trial probabilistic expectation of stop trials and response errors in the stop-signal task, based on a Bayes-optimal sequential estimation (Ide et al., 2013). This work further demonstrates that the response of the ACC is dynamic.

### Salience and the salience network

Salience is a feature of individual objects relating to their appearance frequency within a context or sequence of objects, and so can be quantified by its surprise value. In Bayesian schemes the optimization of precision is seen as an attentional process; however, salience is a bit more complicated. Operationally, salience may be better conceived as a sampling of information that reduces uncertainty. This is fundamentally different from reducing the uncertainty over options—it reduces the uncertainty over states of the world. Under the notions of Itti and Baldi (2009) about Bayesian surprise, salience is quintessentially a measure of the relative entropy or reduction in uncertainty. Something that is surprising is not in itself salient. It is salient by virtue of the fact that it could resolve uncertainty about competing explanations.

The salience network has been proposed to explain the function of the ACC and AI in a new theoretical framework (Menon and Uddin, 2010). It is surprise that drives the activation of the ACC, AI, and associated regions in the frontoparietal network for cognitive control. New models of the ACC have implicitly applied information theory principles. For example, perceived error likelihood is associated with uncertainty but not response conflict. The event-level surprise is positively associated with the predictive error signals from the single-trial level. Decrease of ACC activity during trial-and-error learning is due to reduction of uncertainty. The quantification of salience can also be extended to affective and social domains (Fan et al., 2011). For example, fear is a low frequency event in daily life and has a high surprise value when it occurs.

## Cognitive control architecture

How is cognitive control achieved? In this section, I will examine the levels of cognitive control from Marr's (1982) point of view of complex systems and then argue that the implementation of cognitive control occurs via attentional functions and networks involving frontoparietal regions of the brain for uncertainty reduction.

### Levels of information processing for cognitive control

To understand the architecture of cognitive control, it is useful to consider Marr's model of complex systems (Marr, 1982), which posited three levels of analysis. The first level is the computational theory, which characterizes the problem that a system solves and the principles by which its solution can be computed from the available inputs in natural environments. The computational goal of cognitive control is to optimize uncertainty so that it provides a sufficiently precise action selection and yet properly accommodates irreducible uncertainty about what is knowable in the conflict situation. It is certainly the case that minimizing free energy is predicated on minimizing uncertainty or surprise. The second level is for the representation and algorithm. It describes the procedures executed to produce this solution and the representations or data structures over which the algorithms operate. The coding of inputs and the potential algorithms performing the uncertainty reduction, e.g. in the MFT, the exhaustive search, self-terminating search, and the grouping search algorithms (Fan et al., 2008a), belong to this level. The third level is the hardware implementation that specifies how the algorithms and data structures at the second level are instantiated in the circuits of a brain or a machine. The regions of the frontoparietal network dynamically interact to incorporate the functions of cognitive control, and by using computational modeling and fMRI studies in tasks such as the MFT, the hardware implementation of cognitive control in the brain can be mapped (Wang et al., 2011; Fan et al., 2014).

In one higher-level cognitive architecture model that involves adaptive control of thought, the role of the ACC is goal control—to regulate the internal level of cognition and maintain the task goal, and to allocate the cortical modules of mental operations (Anderson et al., 2008). Activity in the ACC reflects the update of control information. The goal control in the model actually involves channel switching, to coordinate the interaction of several relatively independent modules/processes.

### The implementation of cognitive control via attentional functions and networks

The human body transmits 11 million bits of information per second (bps) to the brain, but our conscious mind can only process a portion of this capacity. For example, reading capacity is estimated as 50 bps, for a typical reading speed of 5 words per second, assuming an average of 5 characters per word and roughly 2 bits per character (Information theory: Applications of information theory: Physiology); visual attention can select only 30–60 bits of information for processing with each glimpse (Verghese and Pelli, 1992). Therefore, cognitive control needs to be involved. Cognitive control is a set of processes that permits adaptive responses consistent with goals and homeostatic demands to constrain the amount of information that reaches focused consciousness. Cognitive control is most needed when there is competition for limited resources and when there is a considerable amount of computation required to determine the most appropriate input and response.

**Figure 5:**
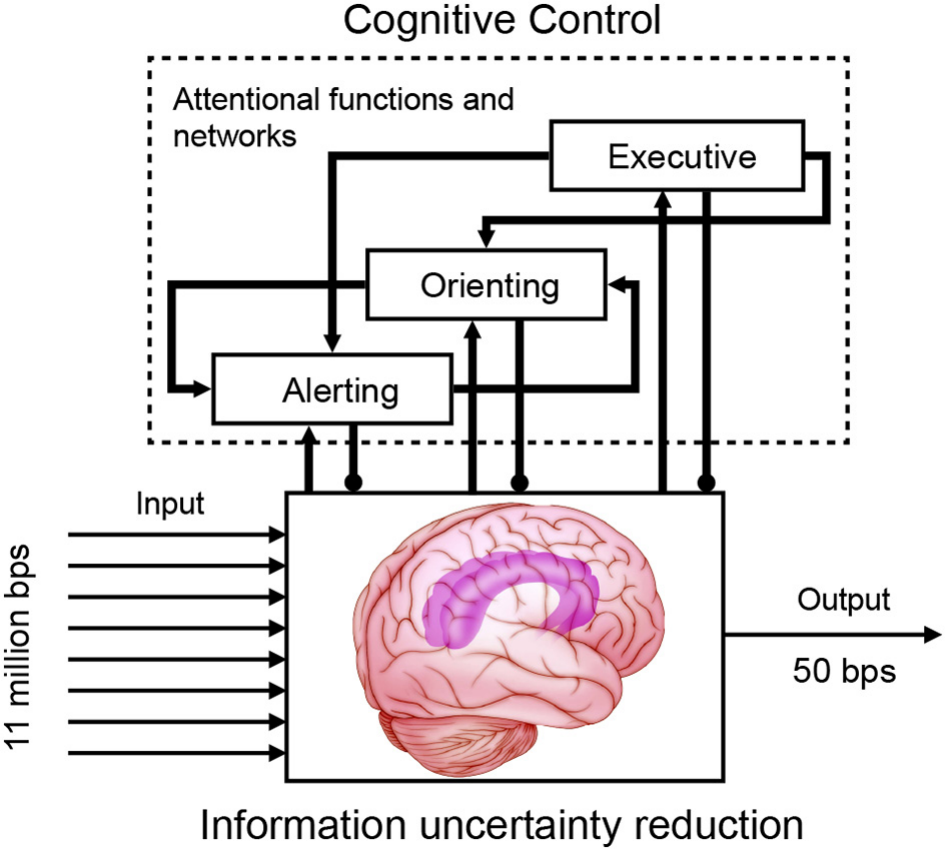
**Cognitive control implemented via attentional functions of alerting, orienting, and executive control**.

It is proposed that cognitive control is implemented by computational mechanisms of distinct and integrated attentional networks which influence information processing for uncertainty reduction (Mackie et al., 2013) (Figure 5). Attention is defined as the activity of a set of brain networks of alerting, orienting, and executive control that influences the priority of computations of other brain networks for access to consciousness or to output (Fan et al., 2002, 2005; Fan and Posner, 2004). Alerting is for achieving an alert state (tonic) and the ability to prepare for a sensory signal (phasic). Orienting is for the selection of information from sensory input and turning attention toward a sensory signal (reflexive or voluntary, covert or overt). Executive control detects and resolves conflict and selects one dimension in the presence of competing information or computation. The coordination of the attentional networks, with modality-independent executive control at the top hierarchical level (Spagna et al., in preparation), functions similar to Normal and Shallice's supervisory attentional system (Norman and Shallice, 1986), and dynamically implements cognitive control in a context-sensitive fashion. It is the mechanism of so called selective attention to deal with the limited capacity of information processing via selectivity (Desimone and Duncan, 1995). This cognitive control architecture is consistent with a key principle of the brain (Friston and Stephan, 2007), with lower regions for sensory input, modulated by alerting and orienting, and higher regions performing multimodal (or association) functions coordinated by executive control.

The neurocomputational architecture of cognitive control is far more complex than what we can plot with a simple flow chart. An increase in uncertainty is related to an increase of demand for cognitive control, which activates not only the ACC and DLPFC, but also distributed regions, such as AI, FEF, and IPC across the frontoparietal network as a function of uncertainty (Fan et al., 2014). Additionally, task and modality specific brain regions and networks should also be modulated by and interact with the frontoparietal network. Further investigation of the functional specification, e.g., the dissociation of the ACC and AI (Gu et al., 2010, 2013a) and integration (Dosenbach et al., 2008) of the cortical and subcortical structures in the frontoparietal network for uncertainty reduction, is needed. This enterprise can begin with an understanding of the mental algorithms (Fan et al., 2008a; Wang et al., 2011) underlying mental processing in cognitive control tasks, i.e., cognitive load or task difficulty, and finding dissociations in regional activity in relation to different aspects of these algorithms (Fan et al., 2014).

## Applications of the information theory account in neuroimaging and clinical studies

### Potential issues in using cognitive subtraction

Beyond addressing the limitations of current theories of cognitive control, this information theory account also addresses other more general issues in the field of cognitive neuroscience. First, despite the well-accepted concept that the brain is comprised of information processing entities, there have been few attempts to quantify this information, which would allow for a quantitative appreciation of the relationship between the input information and neural activity in higher-level systems. Second, although there is a vast literature on computational fMRI using information theoretic constructs and similar computational explanatory variables from control theory and re-enforcement learning, in many fMRI studies researchers compare brain activity between task conditions and make inferences based on the concept and assumptions of cognitive subtraction. The logic behind these inferences supposes that a difference in a property of interest across task conditions accounts for the observed change in neural activation. However, the amount of information across the two task conditions is practically never quantified, and could be an important confounding factor. The difference in activation between two conditions may actually be due to a difference in computational load quantified as information entropy, or due to differences in event probability associated with the surprise. This may explain why common activation in areas such as the ACC (and AI) has been found in different studies using various tasks (Nee et al., 2007; Yarkoni et al., 2011). To address these issues, the information theory framework is needed to move toward a new direction of conducting and understanding studies on cognitive control.

### Information processing deficits in psychiatric disorders

ACC activation abnormalities have been observed in many psychiatric patient populations (Bush et al., 2003). Various cognitive assays known to reliably and robustly activate the ACC have been used to demonstrate aberrant ACC activity and ACC-dependent behavioral performance in patient populations with psychiatric disorders including those with schizophrenia (Carter et al., 2001; Kiehl and Liddle, 2001; Heckers et al., 2004; Mulder et al., 2008; Polli et al., 2008), attention deficit/hyperactivity disorder (Swanson et al., 1998; Bush et al., 1999; Durston et al., 2003; Schulz et al., 2004; Rubia et al., 2005; Mulder et al., 2008), autism (Rinehart et al., 2001; Gomot et al., 2006; Kennedy et al., 2006; Luna et al., 2007; Solomon et al., 2008; Fan et al., 2012), depression (Williams et al., 1996; George et al., 1997), obsessive compulsive disorder (Bannon et al., 2002; Gu et al., 2008), anxiety (Mogg et al., 1993; Benkelfat et al., 1995; Simpson et al., 2001; Hirsh et al., 2012) and neurodegenerative diseases (Seeley et al., 2009; Van Dam et al., 2013). Results from these studies have been discussed in terms of deficits in mental functions such as conflict processing, response inhibition, target/novelty detection, and error detection. Taken together, these results suggest that a general disturbance of the ACC affects a range of its information processing functions and, furthermore, that the processing carried out in this region generally serves as a major bottleneck in terms of task performance. The current literature, however, lacks a comprehensive model that can explain the findings of various ACC-related deficits both within and across patient populations. Additionally, current theories often examine the ACC independently from the frontoparietal network.

In patients with autism, performance was impaired at a fast presentation rate in a Go/No-Go task (e.g., Raymaekers et al., 2004), suggesting that deficits are more detectable under high information processing rates. Patients with autism also show performance deficits on a version of the continuous performance task (Corbett and Constantine, 2006), which is a variation of the Go/No-Go task. These tasks involve an explicit manipulation of stimulus *surprise*, but the nature of their response requirements prevents the measurement of the behavioral effect of entropy. One particular benefit of the information theory account is that it lends itself to the development of a series of cognitive tasks in which information entropy and surprise, computational load, and processing rate can each be systematically manipulated and quantified to examine the relationship between these measures and performance in patient populations (Mackie and Fan, under review). Following a thorough study of the relationship between uncertainty and the frontoparietal network, the information theory account of cognitive control may guide the study of the dysfunction in cognitive control consistently observed in various patient populations and lead to more nuanced predictions.

## Outstanding questions and concluding remarks

### Outstanding questions

There are some additional outstanding questions that need to be answered. First, is the goal of cognitive control a complete reduction of uncertainty, or only to reduce it to a “manageable” level so that we can react appropriately? I would argue that total reduction of uncertainty is not adaptive or even optimal. This may be because a residual degree of uncertainty acts to stimulate vigilance, to handle new, unexpected challenges. Reduction of uncertainty also requires time, and total reduction requires more time than is feasible in experimental or real-world tasks. Indeed, free energy formulations of choice behavior place a great emphasis on the existence of optimal levels of precision (that may be encoded by dopamine) in nuancing the uncertainty about choices in conflict situations (i.e., choice under uncertainty). This suggests that there is indeed an optimal (irreducible) degree of uncertainty that has to be estimated by systems such as the ACC—a perspective that may illuminate the functional anatomy of this region.

Second, how do we demonstrate that ACC activation is related to the amount of information to be processed, rather than prolonged RT *per se*? Empirically, conditions high in uncertainty also have high task difficulty and are accompanied by longer RT. ACC activity related to uncertainty could also be explained simply by the longer time to make responses in a given task (Grinband et al., 2011), rather than task conditions. Studies that isolate ACC activity as a function of uncertainty and hold RT constant are needed to address this issue.

Third, how can we apply information theory to the study of higher-order domain-specific information processing? How can we quantify information processing in other cognitive and emotional processing domains? Information in Shannon's formulation is independent of semantic meaning, and depends entirely on presentation probabilities. However, given the recent focus on Bayesian formulations, one can recast information theory in terms of inference that has an explicit representational (semantic) meaning. This allows us to use the formal constructs of information theory to understand behavior in terms of beliefs and inference. Furthermore, these formal imperatives can be associated with message passing in the brain using schemes like Bayesian filtering and predictive coding. If events in a given domain also differ in their expected probabilities, information uncertainty could be a viable alternative explanation, one that does not rely on domain-specific semantic explanations. For example, fear is often related to activity in the amygdala. However, the contexts that induce fear are usually unlikely to occur, and activity related to fear may be attributed to the high surprise resulting from a low-probability event, or temporal uncertainty (e.g., Herry et al., 2007). The information theory account may provide the tools to quantify processing in these higher-level domains. Testing the interaction of the frontoparietal network with domain-specific regions, i.e., studying domain-specific modulation of cognitive control (e.g., Esterman and Yantis, 2010), will ultimately lead to a more complete understanding of these high-level functions.

### Concluding remarks

The brain is built to cope with uncertainty (Bach and Dolan, 2012). I believe that uncertainty reduction is the goal of cognitive control and that cognitive control emerges as a high-level response to information uncertainty, rather than automatic low-level sensory processes. Information theory lends itself to a quantitative analysis of related brain activation, and understanding the neural bases underlying cognitive control would be a major achievement in cognitive neuroscience, and has great potential to inform basic science as well as to provide a template for understanding the neural basis of mental disorders. Extending tests of the account to clinical populations can also reveal new insights. By conducting lesion studies, we can investigate the necessity of specific regions in the frontoparietal network for cognitive control, as well as their roles and interactions within the system (e.g., Anderson et al., 2008). In addition, we can apply this account to patient populations with cognitive control deficits, such as individuals with autism, in order to better understand the underlying deficits in terms of uncertainty processing.

The goal of this paper is to advance and examine a general and quantitative information theory framework that accounts for cognitive control. This account challenges dominant views of ACC as being important exclusively for conflict monitoring or conflict resolution and offers an alternative with a theory relating uncertainty (or information processing) to ACC activity, as well as activity in the frontoparietal network. Such re-conceptualization could help to push the field forward by providing novel theoretical, methodological, and practical insights. Furthermore, it could lead to the wider adoption of an information theory approach in the field of cognitive neuroscience. As pointed out 55 years ago by Attneave (1959) and reflected in a recent popular science book of Gleick (2011), “information theory is not going to provide a ready-made solution to all psychological problems,” but, “employed with intelligence, flexibility, and critical insight, information theory can have great value both in the formulation of certain psychological problems and in the analysis of certain psychological data.”

### Conflict of interest statement

The author declares that the research was conducted in the absence of any commercial or financial relationships that could be construed as a potential conflict of interest.
